# Could the Extent of Lymphadenectomy Be Modified by Neoadjuvant Chemotherapy in Cervical Cancer? A Large-Scale Retrospective Study

**DOI:** 10.1371/journal.pone.0123539

**Published:** 2015-04-10

**Authors:** Ting Hu, Xiong Li, Qinghua Zhang, Kecheng Huang, Yao Jia, Ru Yang, Fangxu Tang, Qiang Tian, Ding Ma, Shuang Li

**Affiliations:** 1 Department of Obstetrics and Gynecology, Tongji Hospital, Tongji Medical College, Huazhong University of Science and Technology, Wuhan, P.R. China; 2 Department of Gynecology & Obstetrics, the Central Hospital of Wuhan, Wuhan, P.R. China; 3 Institute for Systems Biology, Seattle, Washington, 98109–5234, United States of America; Rajiv Gandhi Centre for Biotechnology, INDIA

## Abstract

**Background:**

The effect of neoadjuvant chemotherapy (NACT) on topographical distribution patterns of lymph node metastasis in cervical cancer was unknown.

**Methods:**

Patients with FIGO stage IB1-IIB who underwent radical surgery with or without NACT were enrolled (3527 patients). A matched-case comparison design was used to compare the effects of NACT on lymph node metastasis.

**Results:**

We analyzed groups of 167 and 140 patients who were diagnosed with lymph node metastasis in the matched primary surgery group and NACT group, respectively, and no significant difference was observed (p = 0.081). The incidence of lymph node metastasis was significantly decreased in the NACT-responsive group compared to the non-responsive group (18.4% vs. 38.6%, P<0.001). The metastatic rates for every lymph node group also declined in the NACT-responsive group except for the deep inguinal and the para-aortic lymph node groups. Clinical response, deep stromal, parametrial and lymph vascular invasions were independent risk factors for lymph node metastasis in the NACT group. Furthermore, deep stromal invasion and lymph vascular invasion, but not the response to NACT, were independently associated with upper LNM. The number of lymph nodes involved, response to NACT, tumor histology and a positive vaginal margin were independent prognostic factors affecting DFS or OS rates in node-positive patients treated with NACT plus radical surgery.

**Conclusion:**

The frequency and topographic distribution of LNM are not modified by NACT, and clinical non-responders showed more involved LNs. A systemic and extensive lymphadenectomy should be performed in patients treated with NACT plus surgery regardless of the response to NACT.

## Introduction

Over the last two decades, neoadjuvant chemotherapy (**NACT**) in combination with radical surgery has been used to treat cervical cancer to reduce tumor size and extension and to treat micrometastatic disease [1]. Even though it is currently not considered as part of the standard of care according to The National Comprehensive Cancer Network (NCCN), combined NACT and surgery is being used in up to 25% of patients in many parts of the world such as Asia, Italy and South America [2].

Lymph node metastasis (**LNM**) is the most important prognostic factor for cervical cancer patients displaying high risk surgical-pathological factors [3, 4]. Although it is not included in the current 2009 FIGO (International Federation of Gynecology and Obstetrics) staging system, the lymph node status represents a crucial factor for the prognostic characterization and management of cervical carcinoma [5]. Whether NACT has a therapeutic effect on the incidence of LNM is controversial [6, 7]. Moreover, the effects of NACT on the topographical distribution pattern of LNM in the pelvic and the para-aortic areas have not been previously reported. It was known that the incidence of LNM was significantly decreased in NACT responders [8], while the topographical distribution pattern of LNM in these patients was also not reported before. Whether the extent of lymphadenectomy could be modified in the following radical surgery in patients treated with NACT, especially in NACT responders is our main point of interest.

In addition, the number of metastatic lymph nodes involved and other lymph node characteristics, such as the location of nodes, number of involved node sites and node size, may be more indicative prognostic factors for long-term survival [9–11]. In patients undergoing primary surgery treatment (**PST**), the number and location of metastatic lymph nodes affect the long-term survival [10]. While a recent study by Kim et al. reported there was no association between the number of metastatic lymph nodes and the survival of patients treated with NACT plus radical surgery compared to patients treated with PST [12]. Moreover, the prognostic factors in patients with LMN treated with NACT plus radical surgery were not previously investigated.

We conducted a large-scale retrospective study with three major objectives, aimed at evaluating the effects of NACT on the lymph node status. The first aim compared the incidence and topographic distribution pattern of LNM in the pelvic and the para-aortic areas in the NACT-treated group and a group of matched patients who underwent primary surgery, and in the NACT responders and non-responders. The second aim was to investigate the risk factors associated with LNM and upper lymph node metastasis (the common being the iliac lymph node, the para-aortic lymph node or the deep inguinal lymph node) in patients treated with NACT plus radical surgery. The third aim was to evaluate the relationship between the number of lymph nodes and the anatomical sites of metastasis on long-term survival and the clinicopathological variables associated with clinical outcomes in NACT-treated patients with LNM. Upon these results, we will investigate the possibility of modifying the extent of lymphadenectomy in the radical surgery in patients treated with NACT.

## Methods

### Patients

Medical records were reviewed from the cervical cancer database (v1.10), which consisted of clinical data from 10897 patients (*http://clinicaltrials.gov*; NCT01267851). This study was reviewed by the Ethics Committee at the Huazhong University of Science and Technology, and informed consent was obtained from each patient. All of the patients were restaged according to the 2009 FIGO classification [13]. Inclusion criteria for the study were as follows: (1) squamous cell carcinoma, adenocarcinoma or squamoadenocarcinoma; (2) FIGO stage IB1-IIB; and (3) patients who underwent radical surgery with or without NACT from January 2002 to December 2008. Small cell carcinomas and other cancer types were excluded because of distinctive tumor behavior. The patient enrollment flow is shown in **S1 Fig**. Because the incidence and topographical distribution pattern for LNM was associated with the FIGO stage, tumor size and other clinical characteristics, in order to eliminate bias, a matched-case study (1:1) for age at diagnosis, FIGO stage, tumor size and histological type was designed to investigated the effects of NACT treatment on LNM, which was described in our previous study [14].

The following pre-treatment routine procedures were performed in all patients: physical and gynecological examination, biopsy and pathological examination of the uterine cervical tumor, complete blood analysis, chest X-ray, pelvic MRI examination and color ultrasound of the liver and kidney. In the primary NACT group, all patients received cisplatin-based chemotherapy followed by radical surgery, and the chemotherapy regimens are detailed in our previous study. The tumor responses were assessed by MRI, a gynecological examination and examination of surgical specimens according to the WHO criteria [15]. A systemic pelvic lymphadenectomy was performed and included all fatty lymphatic tissues in the obturator, external iliac, internal iliac, common iliac and inguinal areas. Parametrial lymph nodes were removed together with the parametrium, and suspicious para-aortic lymph nodes detected by preoperative imaging or intraoperative palpations were also dissected. To distinguish their anatomical regions of origin, all dissected lymphatic tissues were placed in separate labeled specimen bottles for pathological evaluation after surgery. The mean number of lymph nodes dissected from all enrolled patients was 22.1 (range, 7–76). Fifty-seven of 3533 patients underwent a para-aortic lymph node (PALN) dissection due to suspected metastasis within the node. For the purpose of our study, metastases were defined as macroscopic if the nodes were found to be palpably enlarged in surgical findings with obvious signs of tumor tissue on the cut section or if the pathologist reported the lymph nodes to be macroscopically positive. Enlarged lymph nodes containing only microscopic tumor cell foci were considered to be microscopically positive. **Upper lymph node metastasis (ULNM)** was defined as involving the common iliac lymph node, the para-aortic lymph node or the deep inguinal lymph node, and **lower lymph node metastasis (LLNM)** was defined as involving the pelvic lymph node (excluding common iliac lymph node metastasis).

Adjuvant therapy was performed if the patients had the following risk factors: lymph vascular space invasion (**LVSI**), lymph node metastasis, deep stromal invasion (outer 1/3 layer), parametrial invasion, a positive vaginal surgical margin and poor tumor differentiation. Adjuvant therapies consisted of chemotherapy, radiotherapy or chemo-radiotherapy, and the type of postoperative treatment depended on the patient’s age, general health conditions, response to preoperative therapy and the preferred treatment option of the patient and physician.

### Statistical analysis

Significant differences between treatment groups were assessed by performing a Student’s t-test for normally distributed continuous variable data, and a Mann-Whitney U-test or a Kruskal-Wallis test was performed for non-normally distributed continuous variable data. A Pearson Chi-square test or a Fisher’s exact test with or without linear-by-linear association was performed for the categorical data. A logistic regression model was developed to describe predictors of LNM in patients treated with NACT plus radical surgery. Disease-free and overall survival rates (**DFS** and **OS**) were compared and estimated using the Kaplan-Meier method. Variables that showed a significance level of p < 0.2 in the univariate analysis were selected in multivariate analysis (a logistic analysis and a Cox regression analysis were performed) to minimize erroneous exclusion. Stepwise backward selection procedures (p < 0.15 to enter the study, and p < 0.1 to remain in the study) were used in both the logistic and Cox regression analyses.

All statistical tests were two-tailed, and a P value of less than 0.05 was considered to be significant. All statistical analyses were performed using SPSS software 13.0 (SPSS, Inc., Chicago, IL).

## Results

A total of 3533 patients met the criteria for inclusion in the present study. The mean age at diagnosis was 44 (range, 21–68). A total of 1011 patients were treated with NACT plus radical surgery (primary NACT group), and 2522 patients were treated with primary radical surgery (primary PST group). Using a matched-case design, our study consisted of 705 patients in the matched NACT group and 705 patients in the matched PST group (**S1 Table**).

The median number of involved lymph nodes in the enrolled patients with LNM was 2 (range, 1–21). In the primary NACT group, 227 patients (22.5%) had LNM, with 1 in the para-aortic node area, 4 in both the pelvic and the para-aortic node areas and 222 in the pelvic node area. In the primary PST group, 348 patients (13.8%) had LNM, with 2 in both the pelvic and the para-aortic node areas and 346 in the pelvic node area.

### The topographical distribution pattern of involved lymph nodes in the primary NACT group

According to the FIGO staging system, the incidence of LNM within the primary NACT group was as follows: 75/414 (18.1%) in FIGO stage IB, 67/275 (24.4%) in FIGO stage IIA and 85/322 (26.4%) in FIGO stage IIB. These data demonstrated an increasing and significant trend (linear-by-linear association chi-square test, p = 0.006). The median number of total lymph nodes involved in each FIGO stage was as follows: 1 (range, 1–16) in FIGO stage IB, 2 (range, 1–21) in FIGO stage IIA, and 2 (range, 1–17) in FIGO stage IIB, and a marginal significant difference was observed (Kruskal-Wallis test, p = 0.061). The anatomical distribution of all lymph node groups ordered by the metastatic rate was as follows: the obturator (136/227, 59.9%), the internal iliac (82/227, 36.1%), the external iliac (61/227, 26.9%), the common iliac (32/227, 14.1%), the deep inguinal area (18/227, 7.9%), the parametrial area (7/227, 3.1%) and the para-aortic area (5/227, 2.2%). Metastasis confined to one anatomical node group was observed in 111 patients, and the obturator lymph nodes showed a high incidence of metastasis (60 patients), followed by the internal iliac nodes (25 patients), the external iliac nodes (14 patients), the common iliac nodes (4 patients), the deep inguinal nodes (2 patients), the parametrial nodes (2 patients) and the para-aortic nodes (1 patients). The data for 3 patients were unavailable (missing).

### Comparison of incidence and topographical distribution patterns for metastatic lymph nodes between matched NACT and PST groups

**Fig 1:**
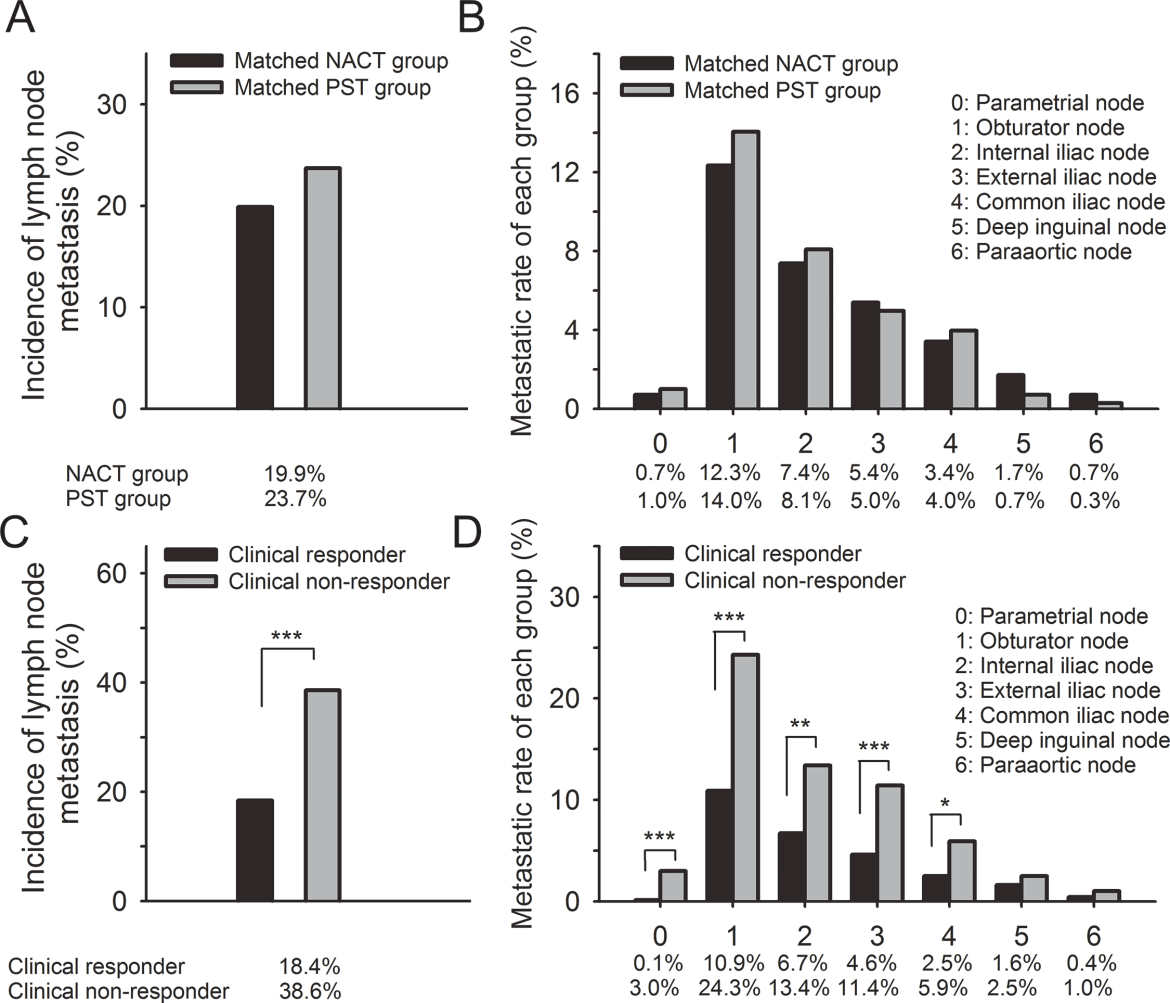
Comparison of incidence and distribution of LNM between the matched NACT and PST group (A-B), clinical responders and clinical non-responders (C-D).

No significant differences were found between the number of lymph nodes removed during surgery between the matched NACT and PST groups (21.6±9.1 vs. 23.1±9.4, respectively, P = 0.119). The number of patients diagnosed with LNM was 167 and 140 for the matched PST and NACT groups, respectively, and no significant difference was observed (p = 0.081). The frequency of metastasis to each lymph node group is shown in **Fig 1.** The obturator, internal iliac and external iliac lymph node groups were the most commonly involved sites in the matched PST and NACT groups. No significant difference was observed when the metastatic rate for every lymph node group was compared between the matched PST and NACT groups (**Fig 1**). Furthermore, stratification of the data based on tumor size, FIGO stage and histology revealed no significant differences between the two treatment matched-groups. However, the FIGO stage IB1-IIA patients and the incidence of squamous cell carcinomas were found to be marginally different between the two treatment groups (P = 0.058 and P = 0.058, respectively; **Table 1**). Solitary LNM was often observed in the obturator, internal iliac and external iliac lymph nodes in both matched PST and NACT groups, and no significant differences between the treatment groups were observed when comparing the topographical distribution pattern for solitary LNM (**S2 Table**). The median number of metastatic lymph nodes involved for cases treated with LNM was 2 (range, 1–16) for the matched PST group and 2 (range, 1–21) for the matched NACT group. Furthermore, no significant differences were observed between the two matched groups for the following: the number of lymph nodes involved, the number of lymph nodes groups involved, lymph node enlargement in node-positive patients and the topographical distribution pattern of involved LNs (**S2 Table**).

**Table 1:** Comparison of incidence of LNM between matched NACT group and PST group, primary NACT group with clinical response and with clinical non-response, respectively, stratified by tumor size, FIGO stage, and histology.

				Primary NACT group		
	Matched PST group	Matched NACT group	P value	Patients with clinical response	Patients with clinical non-response	P value
Total patients	705	705		801	202	
Tumor size						
≤ 4 cm	74/304 (24.3%)	57/304 (18.8%)	0.094	40/297 (13.5%)	33/70 (47.1%)	<0.001
> 4 cm	93/401 (23.2%)	83/401 (20.7%)	0.394	106/498 (21.3%)	45/130 (34.6%)	0.002
FIGO stage						
IB1-IIA	131/577 (22.7%)	105/577 (18.2%)	0.058	92/553 (16.6%)	50/134 (37.3%)	<0.001
IIB	36/128 (28.1%)	35/128 (27.3%)	0.889	55/248 (22.2%)	28/68 (41.2%)	0.002
Histology						
Squamous cell carcinoma	153/638 (24.0%)	125/638 (19.6%)	0.058	128/723 (17.7%)	73/187 (39.0%)	<0.001
Adenocarcinoma	14/67 (20.9%)	7/67 (10.4%)	0.096	19/78 (24.4%)	5/15 (33.3%)	0.467

### Effects of response to NACT on incidence and topographical distribution pattern of LNM

To investigate whether the response to NACT had an effect on the incidence and topographical distribution pattern of LNM, a comparison was made between clinical responders (801 patients) and non-clinical responders (202 patients) within the primary NACT group. Notably, the incidence of lymph node metastasis was significantly decreased in the NACT-responsive group compared to the non-responsive group (18.4% vs. 38.6%, P<0.001) (**S2 Table**). When stratified by tumor size, FIGO stage and histology, significant differences were also observed between the two groups with the exception of adenocarcinomas (P<0.005, **Table 1**). The metastatic rates for every lymph node group involved were significantly decreased in patients who clinically responded to NACT compared to clinical non-responders. However, metastatic rates for the deep inguinal and para-aortic lymph node groups were unchanged (maybe due to the limited sample size, **Fig 1**). When clinical responders were compared to non-responders, the median number of lymph nodes involved, topographical distribution of the LNs involved, number of LN groups involved, LN enlargement in node-positive patients and topographic distribution of solitary LNM remained unchanged (**S2 Table**).

### Risk factors for LNM and ULNM in patients treated with NACT plus radical surgery

The following predictors that were associated with LNM (p<0.20) in our univariate analysis were selected for further analysis: FIGO stage, tumor size, clinical response to NACT, deep stromal invasion status, parametrial invasion status, lymph vascular invasion status and corpus uterine invasion status. In the multivariate analysis, the odds ratios for the independent risk factors for LNM are shown in **Table 2**. Furthermore, only deep stromal invasion and lymph vascular invasion were associated with upper lymph node metastasis in the multivariate analysis, while the response to NACT was not associated with this event.

**Table 2:** Multivariate analyses of risk factors for lymph node metastasis and upper lymph node metastasis in patients who had received NACT.

	OR (95% confidence interval)
***Risk factors for LNM***	
Clinical response to NACT	0.436 (0.303–0.628)
Deep stromal invasion	2.367 (1.695–3.305)
Parametrial invasion	2.310 (1.378–3.870)
Lymph vascular invasion	1.673 (1.037–2.699)
***Risk factors for ULNM***	
Deep stromal invasion	2.871 (1.502–5.485)
Lymph vascular invasion	2.542 (1.222–5.290)

### Survival

**Fig 2:**
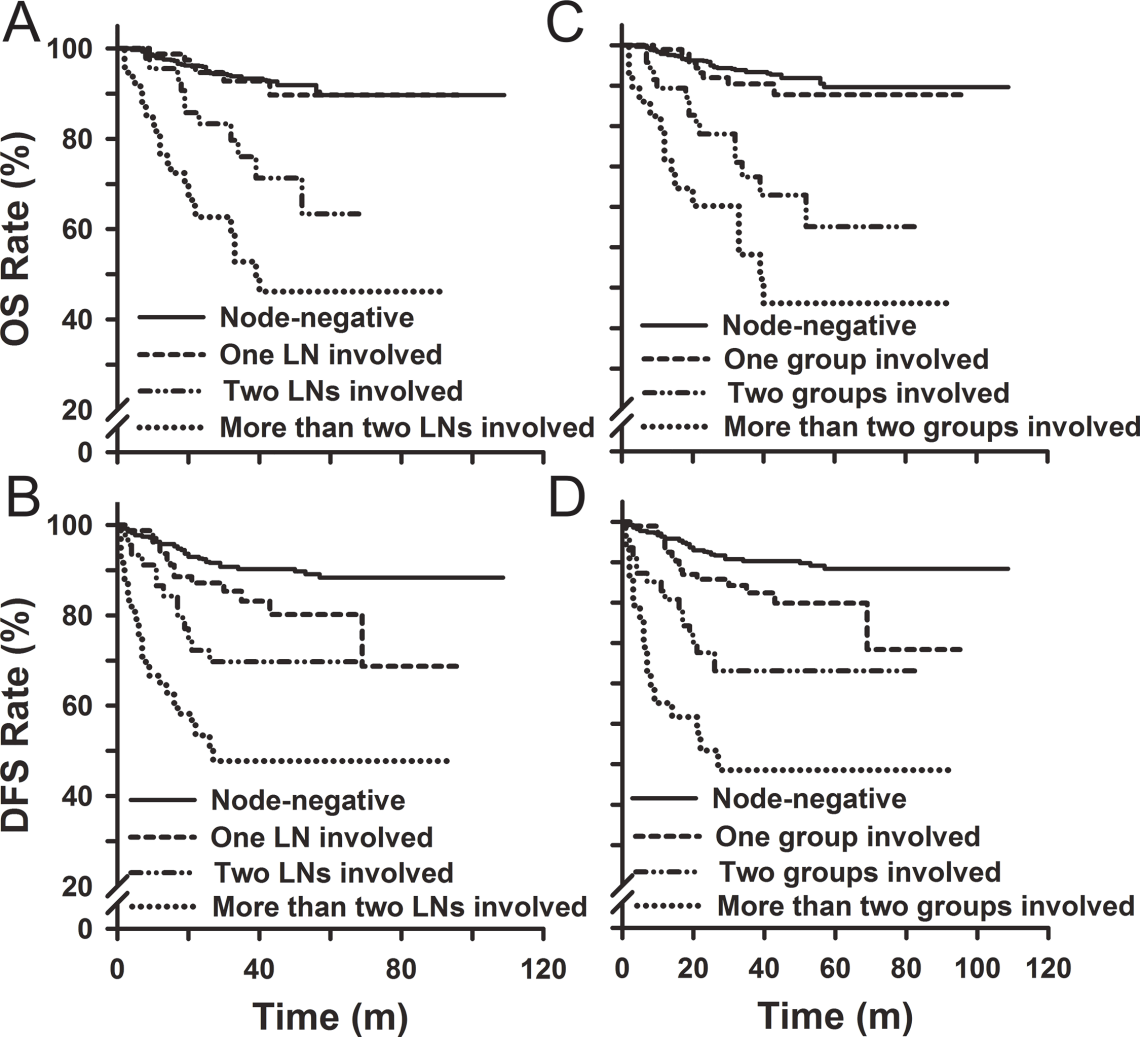
Overall survival and disease-free survival of patients in the primary NACT group by the number of lymph nodes involved (A, B) and the number of lymph node groups involved (C, D).

**Fig 3:**
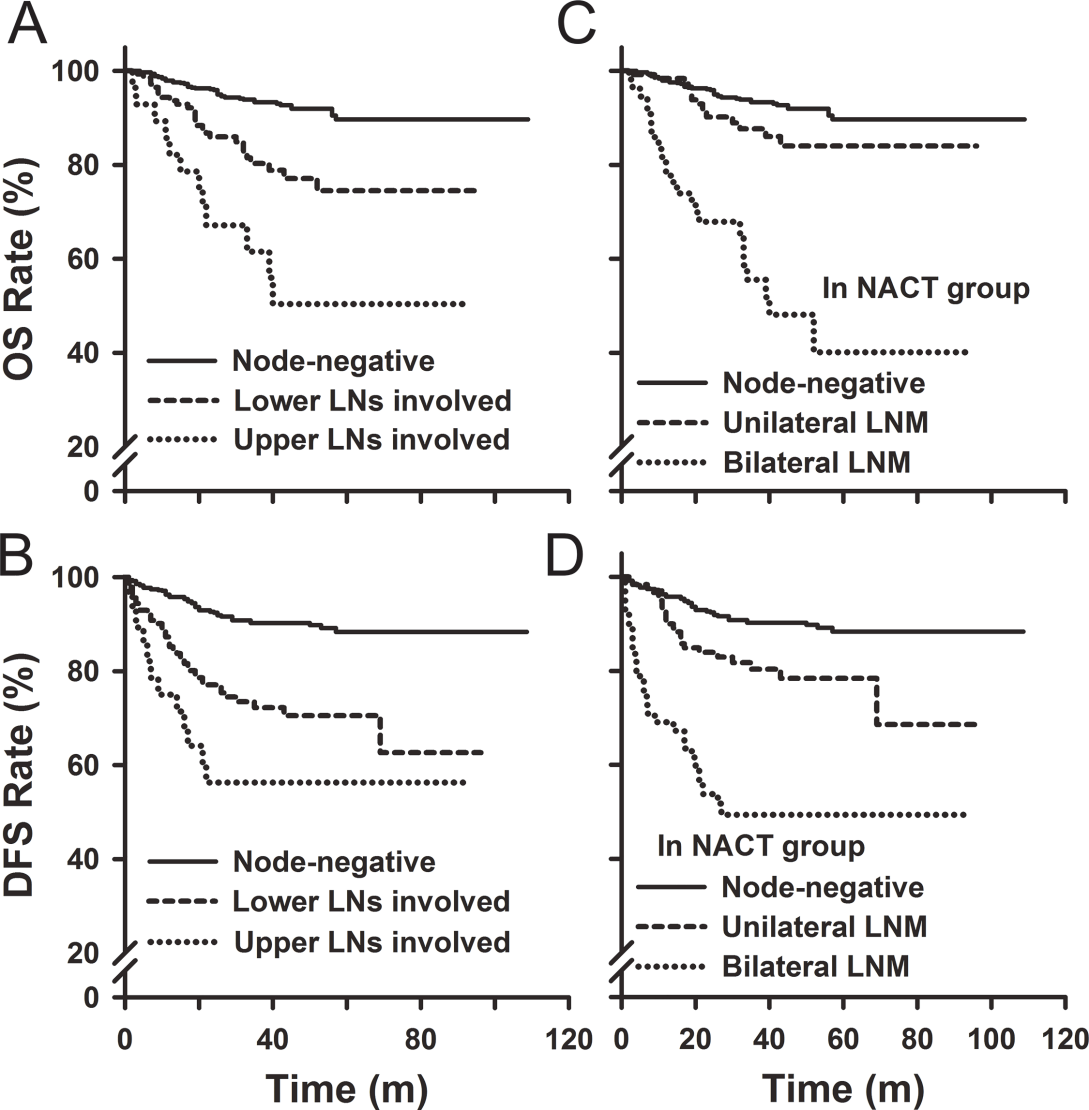
Overall survival and disease-free survival of patients in the primary NACT group by the site of lymph nodes involved. (A-B) negative lymph nodes, lower and upper positive lymph nodes; (C-D) negative lymph nodes, unilateral and bilateral positive lymph nodes.

Among 1011 primary NACT patients, the 5-year DFS rate for patients with one LNM was significantly decreased compared to LNM-free patients (80.2% vs. 88.3%, p = 0.02, **Fig 2**). Meanwhile, one LNM did not alter the 5-year OS rate (p>0.05). Patients with 2 LNs involved showed a lower 5-year DFS rate of 69.7%, and compared to patients with 1 metastatic LN, no significant difference was observed (P = 0.076). However, a significantly lower 5-year OS rate of 63.4% was observed for patients with 2 metastatic LNs compared to patients with one metastatic LN (p = 0.006). Patients with 3 or more metastatic LNs showed a significantly decreased 5-year DFS rate of 47.8% and a 5-year OS rate of 46.2% when compared to patients with either one or two metastatic LNs (p<0.05). The number of involved lymph node sites was also associated with patient outcomes (**Fig 2**). With respect to the sites of LN metastasis, node-positive patients with upper LNM showed a significantly worse prognosis compared to those with lower LNM, and the DFS and OS rates for both patient groups were reduced compared to node-negative patients (P<0.05, **Fig 3**). Similarly, patients with bilateral lymph node metastasis had a worse prognosis compared to those with unilateral LNM, and when compared to node-negative patients, both groups showed lower DFS and OS rates (P<0.05, **Fig 3**). Compared to node-negative patients, the 5-year DFS and OS rates were significantly lower for patients with macroscopic or microscopic LNM (**S2 Fig**). However, no significant difference was observed in the DFS and OS rates between patients with macroscopic LNM and microscopic LNM (**S2 Fig**).

The clinical response to NACT also affected the long-term survival of patients with LNM (**S3 Fig**). Within the primary NACT group, clinical responders with LNM showed a 5-year DFS and OS rate of 73.3% and 74.8%, respectively. Both the DFS and OS rates for clinical responders were significantly higher compared to those for clinical non-responders with LNM (60.4% and 60.8%, p<0.05), and these survival rates were significantly lower than those for patients without LNM (88.4 and 89.7%, p<0.001) (**S3 Fig**).

When examining the multivariate survival analysis in patients with lymph node metastasis who had received NACT (n = 176), the number of metastatic lymph nodes, histology and clinical response to NACT were associated with disease-free survival, while a high number of metastatic lymph nodes, adenocarcinoma or adenosquamous carcinoma, and the presence of a positive vaginal surgical margin were identified as being independent risk factors for overall survival (**Table 3**).

**Table 3:** Multivariate analyses of prognostic factors for patients with lymph node metastasis who had received NACT (n = 176).

	OR	95% confidence interval	P value
***Disease-free survival***			
Number of metastatic lymph nodes			
1	1	reference	
2	2.402	1.113–5.185	0.026
>2	4.789	2.456–9.339	<0.001
Histology			
Squamous cell carcinoma	1	reference	
Adenocarcinoma	3.4	1.701–6.794	0.001
Clinical response to NACT			
Yes	1	reference	
No	1.812	1.024–3.207	0.041
***Overall survival***			
Number of metastatic lymph nodes			
1	1	reference	
2	5.162	1.611–16.540	0.006
>2	15.317	5.178–45.303	<0.001
Histology			
Squamous cell carcinoma	1	reference	
Adenocarcinoma	2.388	0.951–5.997	0.064
Positive vaginal surgical margin			
No	1	reference	
Yes	3.237	1.178–8.891	0.023

The following factors were selected by univariate analysis for survival analysis: age of patient, FIGO stage, histology, tumour grade, LVSI status, tumour size, adjuvant treatment after surgery, deep stromal invasion status, parametrial invasion status, presence of a positive surgical margin, presence of enlarged LNM, clinical response to NACT, the number of metastatic lymph nodes involved, unilateral/bilateral lymph node metastasis, involvement of upper/lower lymph node and the number of metastatic lymph node sites involved.

## Discussion

The therapeutic effect of NACT on LNM is controversial [6–8, 16–20]. Kim et al. proposed the hypothesis that NACT could reduce the mean size of lymph node metastasis and make small metastases difficult to detect [21]. However, we found no significant differences in the rates of lymph node enlargement and the mean number of positive lymph nodes in node-positive patients between the NACT and PST group. Furthermore, in our study, NACT did not appear to reduce the size of metastatic lymph nodes. Generally, the percentage of patients with advanced stage in the NACT group was larger than that in the primary surgery treatment group, which could result in a bias between two groups. We matched (1:1) for FIGO stage between two groups to eliminate the bias [14]. The different results in our study may due to the larger sample size and the different population.

The incidence and topographical distribution pattern observed for LNM patients treated with primary radical surgery have been previously reported, but little information is available regarding patients treated with NACT plus surgery [9, 22–25]. First, in the present study, the obturator lymph node region was found to be the most frequently involved metastatic site within the NACT group, followed by the internal and external iliac lymph nodes, and finally the common iliac lymph nodes. These data agree with the results obtained by Sakuragi and Huang who examined patients treated with primary surgery [9, 22]. There appeared to be no skip metastases of the lymph nodes. Furthermore, subgroup analysis of tumor response to NACT in our study showed that LNM in NACT responders was significantly reduced compared to that in NACT non-responders, which is consistent with findings from two other studies conducted in China [8, 26].

Second, because of a large sample size, for the first time we could examine the effect of NACT on the frequency and topographic distribution of LNM compared to PST treatment in LNM patients. Lymph node metastatic rates in the pelvic or para-aortic lymph nodes for NACT patients were not significantly reduced compared to the matched PST group. However, NACT responders showed fewer involved metastatic lymph nodes in nearly every topographic lymph node site examined (except for the para-aortic node area, perhaps as a result of the limited sample size).

Moreover, the number of lymph nodes involved and the proportion of patients with enlarged lymph nodes were unchanged between the matched NACT and PST groups. It seems that NACT may not affect the tumor load in the involved lymph nodes. This finding is consistent with the explanation of Kim et al., who state that tumor cells within metastatic lymph nodes express high levels of several molecules that promote tumor aggressiveness, such as COX-2 and squamous cell carcinoma antigen, which are also are related to chemo-resistance [6, 27, 28]. Additionally, a lack of response to NACT was shown to be an independent risk factor for LNM in our study; the presence of cervical lesions combined with a lack of response to NACT express high levels of chemo-resistance protein such as COX-2 and squamous cell carcinoma antigen may enhance or be related to LNM [29, 30]. However, our study was limited. Proper evaluation of lymph node enlargement and the sizes of the enlarged lymph nodes were not possible due to the lack of data. Therefore, the effect of NACT on tumor load within the metastatic lymph nodes requires further investigation.

Previously, it was reported that patients treated with primary radical surgery who exhibited macroscopic lymph node metastasis, bilateral node metastasis, multiple positive nodes and had involved common iliac and para-aortic lymph nodes had a worse prognosis compared to patients who exhibited microscopic lymph node metastasis, unilateral node metastasis or had one positive node and positive lymph nodes below the level of the common iliac node [10, 31, 32]. These characteristics of LNM have seldom been reported in patients treated with NACT plus surgery. A recent study by Kim et al. reported that the number of metastatic LNs did not affect the prognosis for the NACT group [12]. Contrary to previous findings, the present study indicated that NACT did not modify the prognostic significance of the number of involved LNs. Moreover, other characteristics of involved LNs, such as unilateral/bilateral LNM, common iliac or para-aortic lymph node involvement and the number of metastatic LN sites also affected the prognosis in the NACT group in the present study. In the multivariate analysis of DFS and OS, only the number of metastatic LNs was an independent prognostic factor for node-positive patients in the NACT group. Among the characteristics of involved LNs, the number of metastatic LNs was the most related to survival outcome. For example, we found that node-positive patients without lymph node enlargement had a comparable prognosis to those with enlarged nodes. Moreover, response to NACT did not alter upper lymph node metastasis, which was independently associated with deep stromal invasion and lymph vascular invasion. Therefore, it is recommended that a systemic lymphadenectomy should be extensively performed, especially with the most frequently involved sites, such as the obturator (136/227, 59.9%), the internal iliac (82/227, 36.1%), the external iliac (61/227, 26.9%), and the common iliac LNs to avoid leaving residual diseased tissue.

## Conclusion

In conclusion, the frequency and topographic distribution of LNM are not modified by NACT, and clinical non-responders showed more involved LNs in nearly every topographical lymph node site examined. A systemic lymphadenectomy should be extensively performed in patients treated with NACT plus surgery regardless of the response to NACT. Moreover, clinical response to NACT, tumor histology, a positive vaginal margin and the number of involved LNs are predictors of clinical outcome in node-positive patients treated with NACT plus radical surgery.

## Supporting Information

S1 Fig Patient enrollment in the present study. (TIF)

S2 Fig Overall survival and disease-free survival of patients in the primary NACT group by the lymph node metastases and the size of the involved lymph nodes (macroscopic/microscopic). (TIF)

S3 Fig Overall survival and disease-free survival of patients in the primary NACT group by the lymph node metastases and clinical response to NACT. (TIF)

S1 Table The clinical and pathological characteristics of patients in the matched NACT and PST group. (DOC)

S2 Table Comparison of characteristics of lymph node involvement between matched NACT group and PST group, NACT group with clinical response and with clinical non-response, respectively. (DOC)
