# Supplementary figures and images for: Could the Extent of Lymphadenectomy Be Modified by Neoadjuvant Chemotherapy in Cervical Cancer? A Large-Scale Retrospective Study
Source: PLoS One. 2015 Apr 10;10(4):e0123539. doi: 10.1371/journal.pone.0123539 (PMC4393094; doi:10.1371/journal.pone.0123539)

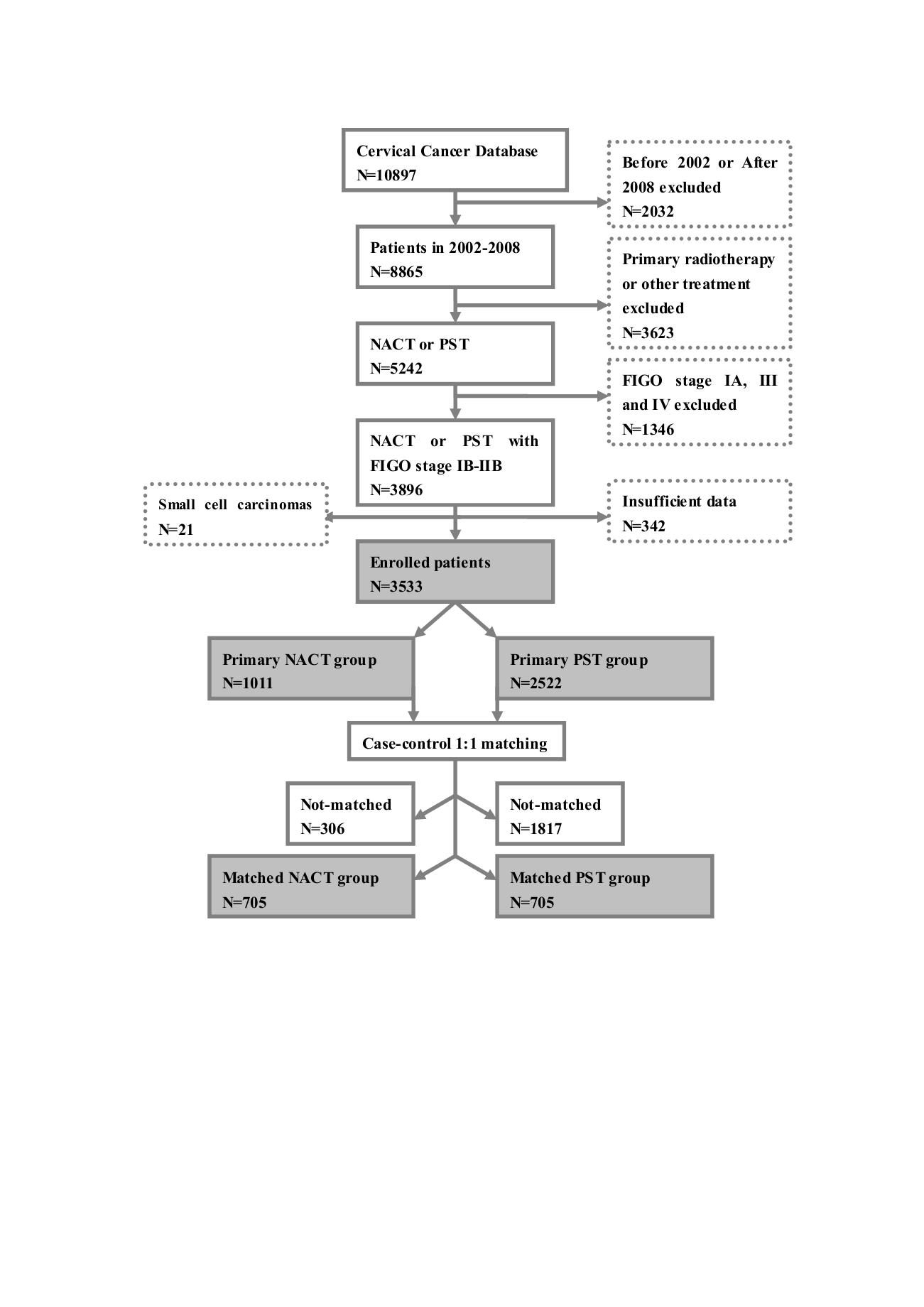

Supplement: S1 Fig — (TIF) [file pone.0123539.s001.tif]

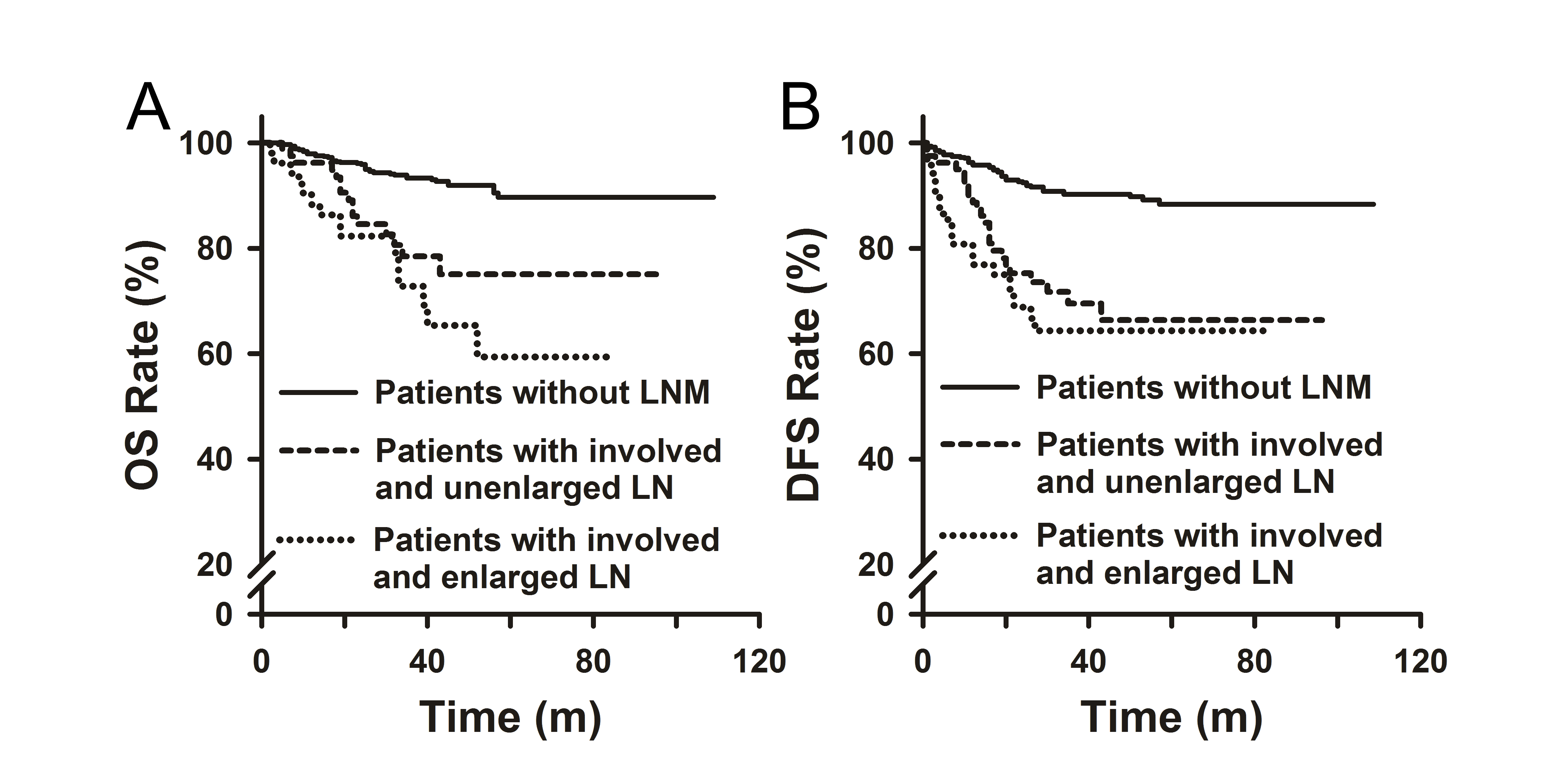

Supplement: S2 Fig — (TIF) [file pone.0123539.s002.tif]

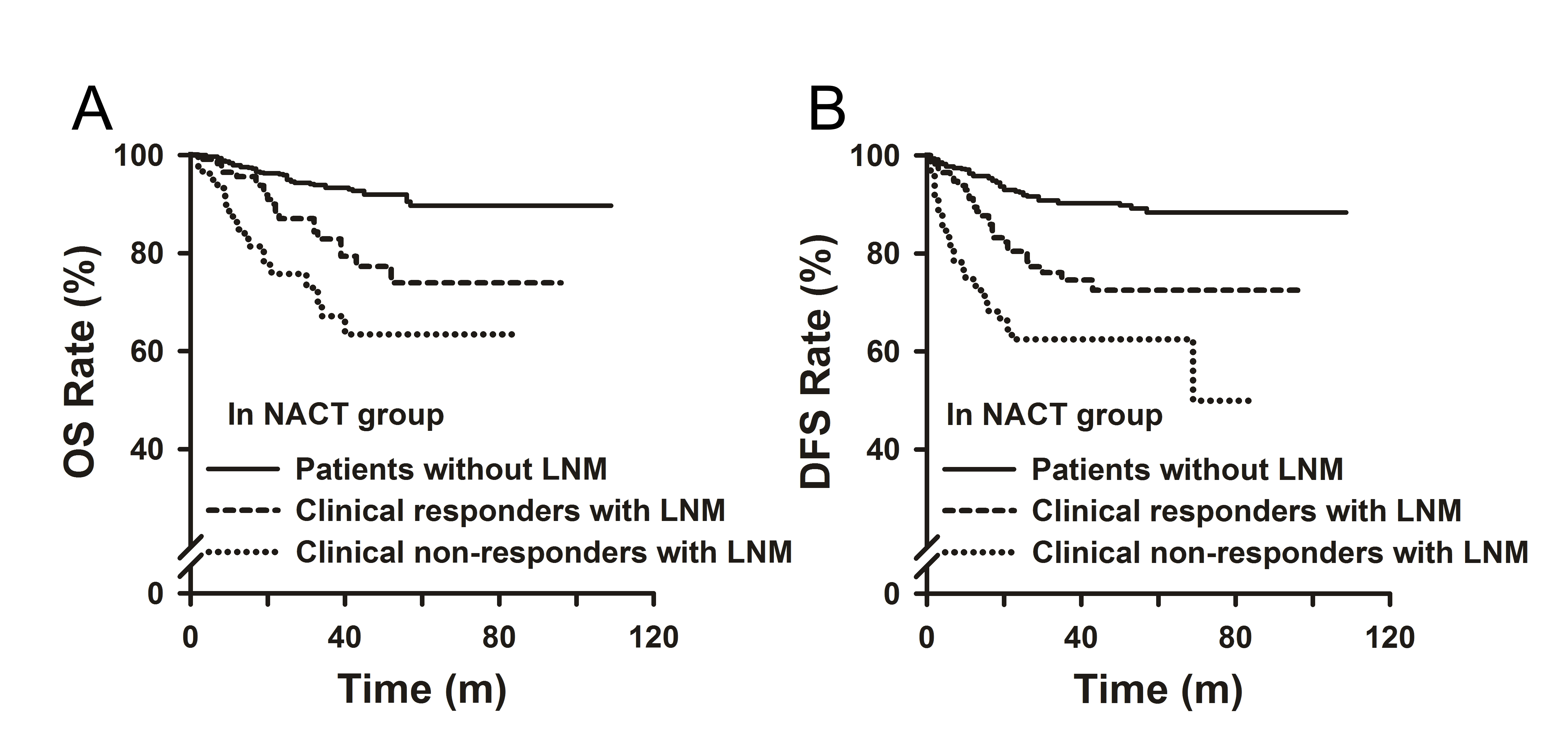

Supplement: S3 Fig — (TIF) [file pone.0123539.s003.tif]
