# Supplementary material for: Could the Extent of Lymphadenectomy Be Modified by Neoadjuvant Chemotherapy in Cervical Cancer? A Large-Scale Retrospective Study
Source: PLoS One. 2015 Apr 10;10(4):e0123539. doi: 10.1371/journal.pone.0123539 (PMC4393094; doi:10.1371/journal.pone.0123539)
Supplement: S1 Table — (DOC) [file pone.0123539.s004.doc]

| **S1_Table.** The clinical and pathological characteristics of patients in the matched NACT and PST group. | | |
| --- | --- | --- |
|  | Matched NACT group | Matched PST group |
| The total number of patients | 705 | 705 |
| Age at diagnosis |  |  |
| Mean ± SD, year | 43.7±8.9 | 43.8±9.0 |
| 20-29 years, n (%) | 33 (4.7%) | 33 (4.7%) |
| 30-39 years, n (%) | 224 (31.8%) | 224 (31.8%) |
| 40-49 years, n (%) | 252 (35.7%) | 252 (35.7%) |
| 50-59 years, n (%) | 156 (22.1%) | 156 (22.1%) |
| 60-69 years, n (%) | 38 (5.4%) | 38 (5.4%) |
| 70-79 years, n (%) | 2 (0.3%) | 2 (0.3%) |
| FIGO stage |  |  |
| IB_1_, n (%) | 126 (17.9%) | 126 (17.9%) |
| IB_2_, n (%) | 204 (28.9%) | 204 (28.9%) |
| IIA_1_, n (%) | 85 (12.1%) | 85 (12.1%) |
| IIA_2_, n (%) | 162 (23.0%) | 162 (23.0%) |
| IIB, n (%) | 128 (18.2%) | 128 (18.2%) |
| Tumor size (cm) |  |  |
| Mean ± SD | 3.7±1.2 | 3.8±1.2 |
| ≤ 2 cm, n (%) | 88 (12.5%) | 88 (12.5%) |
| > 2 cm and ≤ 4 cm, n (%) | 216 (30.6%) | 216 (30.6%) |
| > 4 cm and ≤ 6 cm, n (%) | 390 (55.3%) | 390 (55.3%) |
| > 6 cm, n (%) | 11 (1.6%) | 11 (1.6%) |
| Histological types |  |  |
| Squamous cell carcinoma, n (%) | 638 (90.5%) | 638 (90.5%) |
| Adenocarcinoma, n (%) | 67 (9.5%) | 67 (9.5%) |
| Age at diagnosis, stage, histological types and tumor size between the two groups were well matched. | | |
