# Supplementary material for: Could the Extent of Lymphadenectomy Be Modified by Neoadjuvant Chemotherapy in Cervical Cancer? A Large-Scale Retrospective Study
Source: PLoS One. 2015 Apr 10;10(4):e0123539. doi: 10.1371/journal.pone.0123539 (PMC4393094; doi:10.1371/journal.pone.0123539)
Supplement: S2 Table — (DOC) [file pone.0123539.s005.doc]

| **S2_Table.** Comparison of characteristics of lymph node involvement between matched NACT group and PST group, NACT group with clinical response and with clinical non-response, respectively. | | | | | | |
| --- | --- | --- | --- | --- | --- | --- |
|  | PST group | NACT group | *P* value | NACT group | | *P* value |
|  |  |  |  | Patients with clinical response | Patients with clinical non-response |  |
| Total patients | 705 | 705 |  | 801 | 202 |  |
| Median number of involved lymph node | 2  (range, 1-16) | 2  (range, 1-21) | 0.256 | 2  (range, 1-16) | 2  (range, 1-21) | 0.911 |
| Lymph node enlargement in node-positive patients |  |  |  |  |  |  |
| Macroscopic | 45 (26.9%) | 47 (33.6%) | 0.925 | 44 (29.9%) | 30 (38.5%) | 0.486 |
| Microscopic | 58 (34.7%) | 59 (42.1%) |  | 64 (43.5%) | 35 (44.9%) |  |
| Unknown | 64 (38.3%) | 34 (24.3%) |  | 39 (26.5%) | 13 (16.7%) |  |
| Number of involved lymph nodes |  |  |  |  |  |  |
| 1 | 53 (31.7%) | 56 (40.0%) | 0.224 | 57 (38.8%) | 37 (47.4%) | 0.099 |
| 2 | 41 (24.6%) | 37 (26.4%) |  | 45 (30.6%) | 14 (17.9%) |  |
| >2 | 65 (38.9%) | 43 (30.7%) |  | 40 (27.2%) | 26 (33.3%) |  |
| Unknown | 8 (4.8%) | 4 (2.9%) |  | 5 (3.4%) | 1 (1.3%) |  |
| Number of involved lymph node groups |  |  |  |  |  |  |
| 1 | 67 (40.1%) | 67 (47.9%) | 0.248 | 70 (47.6%) | 39 (50.0%) | 0.324 |
| 2 | 56 (33.5%) | 37 (26.4%) |  | 46 (31.3%) | 19 (24.4%) |  |
| >2 | 31 (18.6%) | 32 (22.9%) |  | 25 (17.0%) | 19 (24.4%) |  |
| Unknown | 13 (7.8%) | 4 (2.9%) |  | 6 (4.1%) | 1 (1.3%) |  |
| Solitary lymph node metastasis |  |  |  |  |  |  |
| Parametrial node | 1 (1.5%) | 0 (0.0%) | 0.541 | 0 (0.0%) | 2 (5.1%) | 0.321 |
| Obturator node | 34 (50.7%) | 34 (50.7%) |  | 40 (57.1%) | 20 (51.3%) |  |
| Internal iliac node | 18 (26.9%) | 15 (22.4%) |  | 16 (22.9%) | 8 (20.5%) |  |
| External iliac node | 5 (7.5%) | 10 (14.9%) |  | 8 (11.4%) | 5 (12.8%) |  |
| common iliac node | 5 (7.5%) | 4 (6.0%) |  | 3 (4.3%) | 1 (2.6%) |  |
| Deep inguinal node | 0 (0.0%) | 1 (1.5%) |  | 2 (2.9%) | 0 (0.0%) |  |
| Paraaortic node | 0 (0.0%) | 1 (1.5%) |  | 0 (0.0%) | 1 (2.6%) |  |
| Unknown | 4 (6.0%) | 2 (3.0%) |  | 1 (1.4%) | 2 (5.1%) |  |
|  | | | | | | |
